# Smoking-Related Multi-Metal Exposure Associated with Hierarchical Gut Microbiota Alterations in Colorectal Polyp Patients

**DOI:** 10.3390/toxics14100902

**Published:** 2026-10-12

**Authors:** Jiali Long, Di Wu, Yingru Liang, Rong He, Yongxian Li, Lei Tan, Peng He, Jun Yuan, Jichuan Shen, Boheng Liang, Shuxian Chen

**Affiliations:** 1 Institute of Public Health, Guangzhou Medical University & Guangzhou Center for Disease Control and Prevention (Guangzhou Health Supervision Institute), Guangzhou 510440, China; 2 Department of Non-Communicable Disease Prevention and Control, Guangzhou Center for Disease Control and Prevention (Guangzhou Health Supervision Institute), Guangzhou 510440, China; 3 Central Laboratory, Guangzhou Center for Disease Control and Prevention (Guangzhou Health Supervision Institute), Guangzhou 510440, China; 4 Department of Pathogenic Microbiology Laboratory, Guangzhou Center for Disease Control and Prevention (Guangzhou Health Supervision Institute), Guangzhou 510440, China

**Keywords:** smoking-related metal exposure, a multi-metal disruption index, gut microbiota, colorectal polyps, hierarchical effects

## Abstract

Smoking-related metal exposure involves complex mixtures, yet its combined effects on gut microbiota and colorectal polyp progression remain unclear. We constructed a multi-metal disruption index integrating urinary arsenic, cadmium, and zinc concentrations that differed significantly between smokers and non-smokers in 196 colorectal polyp patients in 2023-2024 and examined its associations with gut microbiota and colorectal polyp progression. The index showed dose-dependent increases in detection rates of *Limosilactobacillus*, *Methylovirgula* and *Peptoclostridium*, with the most robust association observed for *Limosilactobacillus* after adjusting for covariates (OR = 3.39, 95%CI: 1.49–7.76, *p* = 0.004). Its detection rate was broadly negatively associated with the predicted abundance of pathways such as metabolism and organismal systems. In stratified analyses, the number of pathways with decreased predicted abundance among *Limosilactobacillus*-positive samples increased significantly as index levels rose (low: 0/27; medium: 15/27; high: 19/27), with 10 pathways significant at both medium and high levels. This dose-dependent genus-level response was observed alongside population-level relative stability, as no significant associations were found between the index and colorectal polyp progression. This multi-Metal Index approach provides a practical framework for assessing the health effects of smoking-related multi-metal mixtures. The dose-dependent increase in Limosilactobacillus colonization, occurring together with the decreased predicted abundance of specific metabolic pathways, suggests a possible association with metal-related stress.

## 1. Introduction

Colorectal polyps are the primary precursors to colorectal cancer, with approximately 80% of these malignancies arising from the malignant transformation of adenomatous polyps [1]. In China, colorectal cancer ranks second in incidence among all malignancies, with over 510,000 new cases reported and more than 240,000 deaths annually [2]. The incidence of colorectal polyps is rising and shifting toward younger populations [3]. Cigarette smoking has been firmly established as a significant risk factor for both polyp development [4] and postoperative recurrence [5], yet the molecular mechanisms linking smoking to polyp initiation and progression through intestinal microecology remain incompletely understood.

The gut microbiota has emerged as a key mediator in colorectal polyp progression, with patients showing altered microbial composition compared to healthy controls [6]. Smoking is known to induce gut dysbiosis and impair intestinal barrier function [7]. Among the smoking-induced alterations, heavy metals carried in cigarette smoke may be a critical yet frequently overlooked factor. In smokers, these heavy metals can be directly inhaled and absorbed or ingested via mucociliary clearance [8], leading to bioaccumulation and elevated urinary concentrations [9,10]. Heavy metal exposure disrupts gut microbial structure and function, affecting key metabolism pathways [11]. Collectively, these observations suggest that smoking may indirectly affect the intestinal microecology through the heavy metals.

Despite this evidence, studies integrating smoking-related heavy metal exposure, gut microbiota, and colorectal polyp progression remain limited. Most previous research has focused on a single metal, failing to capture the complexity of multi-metal coexposure patterns that reflect real-world exposure scenarios. Furthermore, the metabolic response of essential trace elements to heavy metal exposure and their potential as markers of metal homeostasis disruption have received limited attention. Therefore, we constructed a smoking-related multi-metal disruption index using urinary metal concentrations, moving beyond the single-pollutant approach. We then examined its associations with the gut microbiota across diversity, specific genera, and metabolic pathways and extended this investigation to colorectal polyp progression.

## 2. Materials and Methods

### 2.1. Study Design and Population

This cross-sectional study included patients who underwent colonoscopy at secondary or tertiary hospitals between November 2023 and July 2024 as part of the Guangzhou Colorectal Cancer Screening Program and were pathologically diagnosed with colorectal polyps. Eligible participants provided complete clinical data, urine and stool samples, and signed informed consent. We excluded individuals with a previous or current diagnosis of colorectal cancer or inflammatory bowel disease, as well as pregnant or lactating women and children.

### 2.2. Data Collection

Trained interviewers administered a standardized questionnaire to collect sociodemographic information, lifestyle behaviors, dietary history, and medical history. Body mass index (BMI) was calculated from measured height and weight. Smoking status was categorized as never smoker, current smoker, or former smoker.

### 2.3. Exposure Assessment

Urinary metal concentrations: midstream urine samples (10 mL) were collected before colonoscopy, transferred into metal-free cryovials, and stored at −80 °C until analysis. At the Guangzhou Center for Disease Control and Prevention laboratory, concentrations of 26 metals (Ag, Al, As, Ba, Be, Cd, Co, Cr, Cs, Cu, Fe, Ga, Li, Mn, Mo, Ni, Pb, Rb, Sb, Se, Sn, Sr, Tl, U, V, and Zn) were measured using an inductively coupled plasma-mass spectrometry (ICP-MS, Model 7900, Agilent Technologies, Santa Clara, CA, USA). Blank controls and standard reference materials were included for quality control. Recovery rates for all target metals ranged from 80% to 120%. Metal concentrations are reported in μg/L, and values below the limit of detection were replaced with one-tenth of the detection limit.

Index of smoking-related multi-metal disruption: The Metal Index was constructed by integrating the concentrations of smoking-associated metals into a single composite measure. Smoking-associated metals were selected from 26 measured urinary metals using a two-step criterion. First, metals with significant concentration differences across current, former, and never smokers were identified (*p*_adj < 0.05). Second, these metals were further examined in pairwise comparisons. Metals showing significant differences in both current vs. never smoker and current vs. former smoker comparisons (*p*_adj < 0.05) were considered smoking-associated. Metals that were significant in the current vs. never smoker comparison but lost significance in the current vs. former smoker comparison were regarded as potentially influenced by smoking cessation and were excluded. Metals meeting both criteria were considered smoking-associated and included in the calculation of the Metal Index. Log-transformed concentrations of these metals were standardized to Z-scores. Following the weighted linear combination principle, the Metal Index was computed as a weighted sum of these standardized concentrations. Weights were derived from regression coefficients for smoking status in models adjusted for age, sex, BMI, alcohol consumption, and tea drinking habits, and were normalized to sum to 1. Higher index values reflect greater smoking-related metal disruption burden.

### 2.4. Outcome Assessment

Stool sample collection and 16S rRNA gene sequencing: Fresh stool samples (approximately 5 g) were collected before colonoscopy, placed in sterile cryovials, and stored at −80 °C until processing. Total DNA was extracted using the E.Z.N.A.^®^Stool DNA Kit (Omega Bio-tek, Norcross, GA, USA). DNA integrity was assessed by 1% agarose gel electrophoresis (Thermo Fisher Scientific, Waltham, MA, USA), and concentrations were quantified using a Qubit^®^3.0 fluorometer (Thermo Fisher Scientific, Waltham, MA, USA). The V4-V5 region of the bacteria 16S rRNA gene was amplified by PCR using primers 341F (5′-CCTAYGGGRBGCASCAG-3′) and 806R (5′-GGACTACNNGGGTATCTAAT-3′). PCR products were purified using the AxyPrep DNA Gel Extraction Kit (Axygen Biosciences, Union City, CA, USA). Purified amplicons were used to construct an Illumina Pair-end library, and paired-end sequencing (2 × 300) was performed on an NGS platform (Shanghai BIOZERON Biotech. Co., Ltd., Shanghai, China).

Bioinformatics analysis: Raw FASTQ files were quality-filtered using Trimmomatic (version v0.41) to remove low-quality reads and those containing ambiguous characters. The DADA2 algorithm (version 1.18.0, QIIME 2 recommended) was used for read trimming, merging, and chimera filtering to generate an amplicon sequence variant (ASV) feature table. Rarefaction curves were then constructed to assess whether sequencing depth adequately captured microbial diversity. Taxonomic assignment of ASVs was performed against the SILVA (SSU138.1) 16S rRNA database with an 80% confidence threshold. Raw sequence counts in the ASV table were converted to relative abundances to account for differences in sequencing depth across samples. A genus was defined as “detected” if its relative abundance was greater than 0 in at least 5% of samples. The PICRUSt2 (version 2.5.3, Phylogenetic Investigation of Communities by Reconstruction of Unobserved States) program, based on the KEGG (Kyoto Encyclopedia of Genes and Genomes) database, was used to predict the functional alteration of microbiota in different samples. We extracted pathway abundance data at Level 2 for subsequent analyses.

Colorectal polyp progression assessment: Participants were classified into three groups based on colonoscopy and pathological findings, following consensus guidelines and pathological diagnostic criteria [12]: non-adenomatous benign lesions, non-advanced adenomas, and advanced adenomas. Advanced adenomas were defined as meeting any of the following criteria: adenoma ≥10 mm, villous or tubulovillous histology, or presence of high-grade intraepithelial neoplasia. Non-advanced adenomas were defined as adenomas with a diameter <10 mm without villous or tubulovillous histology or high-grade intraepithelial neoplasia. Benign polyps that did not meet the pathological criteria for adenomatous lesions were classified as non-adenomatous benign lesions.

### 2.5. Statistical Analysis

Trained researchers used WPS Office for data entry and verification. All statistical analyses were performed using R version 4.3.3. Categorical variables were presented as frequencies (*n*, %) and compared between groups using the chi-square test or Fisher’s exact test. Continuous variables were presented as medians with interquartile ranges and compared using the Mann–Whitney U test or the Kruskal–Wallis test, as appropriate. We calculated variance inflation factors (VIFs) for the three log-transformed metal concentrations to confirm the absence of significant collinearity (VIF < 5). Alpha diversity was compared across groups using the Kruskal–Wallis test, and beta diversity was assessed using PERMANOVA based on Bray–Curtis distances. Trend analysis was performed using the Cochran–Armitage trend test. Associations between genus detection rates and the Metal Index were assessed using multivariate logistic regression with progressive covariate adjustment in three models: unadjusted, smoking-adjusted, and fully adjusted (including smoking, age, sex, BMI, alcohol consumption, and tea drinking habits). Associations between genus detection rates and KEGG pathway abundances were evaluated using the Wilcoxon rank-sum test. Multiple comparisons were corrected using Benjamini–Hochberg false discovery rate (FDR) method. All FDR-corrected *p*-values are reported as *p*_adj, and *p*_adj < 0.05 was considered statistically significant after correction for multiple comparisons. For pairwise comparisons that did not require multiple-comparison correction, a two-sided *p* < 0.05 was considered statistically significant.

## 3. Results

### 3.1. Study Population and Baseline Characteristics

A total of 214 patients with colorectal polyps confirmed by colonoscopy were identified between 1 November 2023 and 31 May 2024. Of these, nine were excluded due to missing 16S rRNA sequencing data, two due to missing urinary metal data, and seven due to a history of intestinal surgery within 6 months before enrollment. The final analytic cohort consisted of 196 patients.

The baseline characteristics of the final cohort are summarized in Table S1. Participants were categorized by smoking status into never smokers (*n* = 138), current smokers (*n* = 34), and former smokers (*n* = 24). Of the 196 patients, 11 (5.5%) had non-adenomatous benign lesions, 144 (72.4%) had non-advanced adenomas, and 44 (22.1%) had advanced adenomas.

### 3.2. Urinary Metal Screening and Construction of the Metal Index

To identify urinary metals associated with smoking status, concentrations of 26 urinary metals were compared across the three smoking-status groups (Table S1). As, Cd, Sr, and Zn showed significant differences and were further examined in pairwise comparisons. Among these, As, Cd, and Zn showed significant differences both between current and never smokers and between current and former smokers (*p*_adj < 0.05). They were therefore considered smoking-associated and retained for the calculation of the Metal Index. In contrast, Sr was significant in the current vs. former smoker comparison (*p*_adj = 0.039) but lost significance when current smokers were compared with never smokers (*p*_adj = 0.068), and was therefore regarded as potentially influenced by smoking cessation and excluded. Median concentrations of all three retained metals were highest in the current smoker group, while the former smoker and never smoker groups showed similar levels (both *p*_adj > 0.05), suggesting normalization after smoking cessation.

VIF for the three metals were all below 2, indicating no collinearity among them. These three metals were thus included together in the index construction. Former and never smokers were combined into a non-smoking reference group, as they did not differ significantly in any of the three metals. After weight normalization, the As, Cd, and Zn weights in the Metal Index were 0.496, 0.327, and 0.177, respectively, with As contributing the most. The index was significantly higher in current smokers than in non-smokers (*p* = 0.003), and positively correlated with each metal (As: r = 0.510, Cd: r = 0.304, Zn: r = 0.536, all *p* < 0.001).

### 3.3. Dose–Response Trends in Genus Detection Rates

Rarefaction curves reached a plateau for all samples, confirming adequate sequencing depth for subsequent analyses. To examine dose–response relationships between the Metal Index and gut microbiota, participants were divided into tertiles of the index: T1 (low, *n* = 65), T2 (medium, *n* = 66), and T3 (high, *n* = 65). Neither alpha diversity (Observed, Shannon, Simpson, and Pielou indices) nor beta diversity differed significantly across the three groups (all *p* > 0.05), indicating no statistical association between the Metal Index and overall gut microbial community structure.

**Figure 1:**
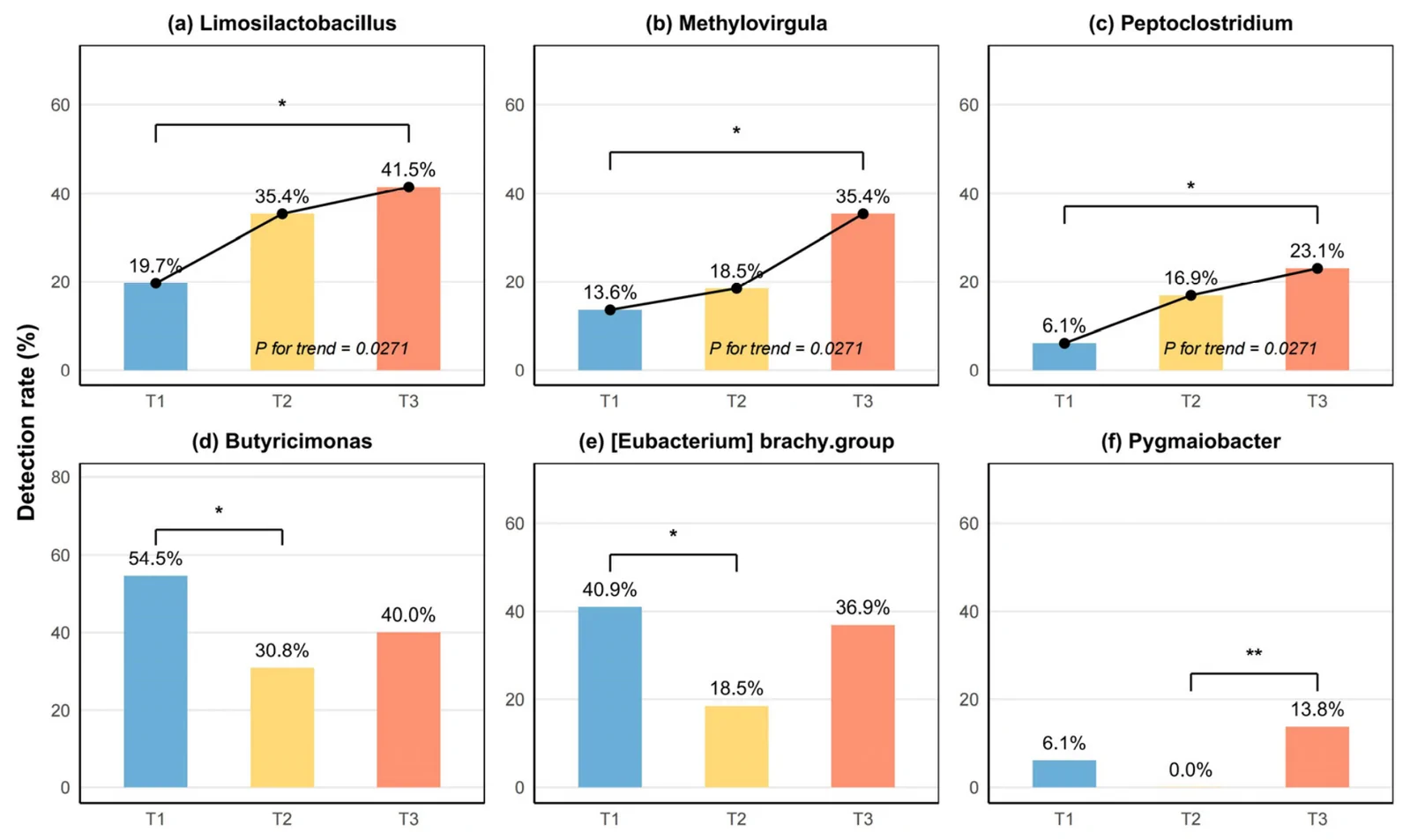
Detection rates of six differential genera across Metal Index tertiles. Bar plots showing detection rates (%) of six genera in T1, T2, and T3 groups. Asterisks indicate significant pairwise comparisons after FDR correction (* *p*_adj < 0.05, ** *p*_adj < 0.01). Solid lines denote significant increasing trends (*p* for trend_adj < 0.05), with trend *P* values shown in the lower right corner of each panel. Among the six genera, *Limosilactobacillus*, *Methylovirgula*, and *Peptoclostridium* exhibited a progressively increasing detection rate with rising Metal Index levels, demonstrating significant dose–response relationships.

At the genus level, however, eleven genera showed significant differences in detection rates across the three groups. After pairwise comparisons and FDR correction, six genera remained statistically significant (*p*_adj < 0.05). Among these, three genera (*Methylovirgula*, *Limosilactobacillus*, and *Peptoclostridium*) showed significant upward trends in detection rates with increasing Metal Index (*p* for trend_adj < 0.05) (Table 1, Figure 1). The detection rate of *Limosilactobacillus* increased from 19.7% in T1 to 41.5% in T3 (*p*_adj = 0.016), which was the highest among the three trending genera in T3.

We next assessed genus relative abundance in two ways, across all genera and specifically among the three trending genera, to determine whether detection rate changes were accompanied by abundance shifts. Of all genera examined, fifteen showed significant differences among groups, but all had low median abundances (<0.59%), and none exhibited a dose–response trend. Among these were the three trending genera, for which pairwise comparisons after FDR correction yielded one significant difference each. However, the relative abundance values were very small, suggesting limited biological relevance. The combined findings from the detection rate and abundance analysis suggest that the Metal Index primarily affects the colonization status of specific genera rather than their abundance after colonization.

The associations between the three trending genera and the index remained independent of smoking and other confounders. Multivariate logistic regression with progressive covariate adjustment (Table S2) showed that ORs for the three genera remained stable and increased across index tertiles after adjustment. *Methylovirgula* had the strongest effect (fully adjusted, T3 vs. T1: OR = 4.67, 95% CI: 1.85–11.82, *p* = 0.001). *Limosilactobacillus* showed the smallest change in OR, indicating a relatively stable association. *Peptoclostridium* effects attenuated but remained significant (fully adjusted, T3 vs. T1: OR = 3.70, 95% CI: 1.10–12.51, *p* = 0.035). In sensitivity analyses without the index, smoking itself was not associated with *Methylovirgula* or *Limosilactobacillus* in models (both *p* > 0.05), but was positively associated with *Peptoclostridium* (OR = 3.32, *p* = 0.025). Together, these findings indicate that the associations between the three genera and the index largely reflect an independent effect of metal disruption rather than serving solely as a proxy for smoking status. *Peptoclostridium* was associated with both smoking and the index, suggesting dual direct and indirect influences.

### 3.4. Genus–Pathway Associations

Colonization by specific genera was associated with metabolic pathway abundance changes. Samples with *Limosilactobacillus* detection had significantly lower abundance in 27 pathways (*p*_adj < 0.05), broadly spanning metabolism, organismal systems, information processing, and cellular processes. Samples with *Methylovirgula* detection had nine associated pathways, with four showing higher abundance (mainly in human diseases) and five showing lower abundance. Samples with *Peptoclostridium* detection showed no significant pathway associations.

**Figure 2:**
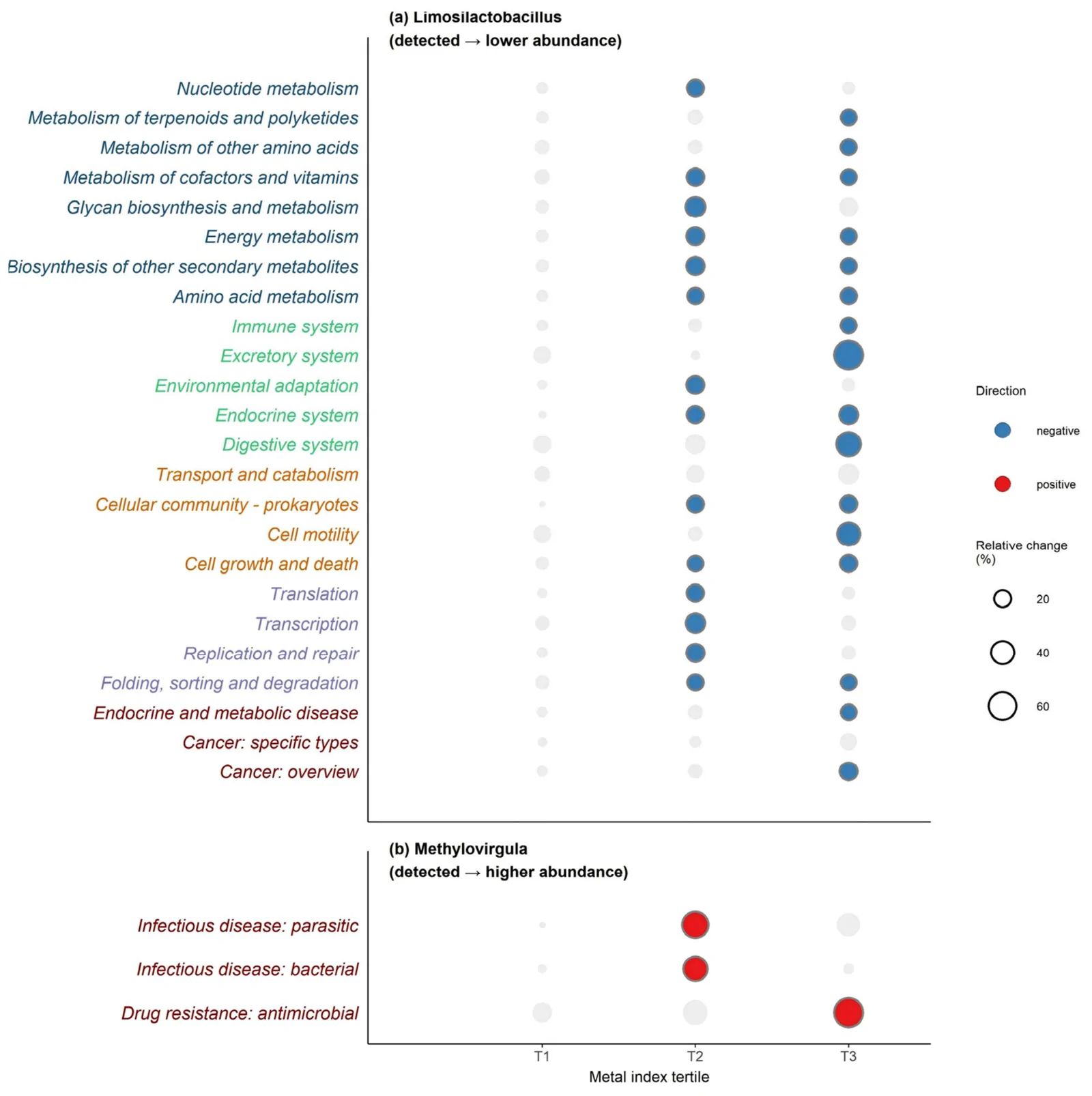
Genus–pathway associations across Metal Index tertiles. This figure shows comparisons of KEGG Level 2 pathway abundance between samples with and without detection of each genus within each tertile. Panels (**a**) and (**b**) show *Limosilactobacillus* and *Methylovirgula*, respectively. *Peptoclostridium* is not shown because it had no significant pathway associations. Point color indicates direction, and point size reflects effect magnitude. Gray points indicate no significant difference (*p* ≥ 0.05), red points indicate significantly higher abundance in detected samples (*p* < 0.05), and blue points indicate significantly lower abundance in detected samples (*p* < 0.05). Point size represents the absolute median difference as a percentage of the non-detected group median, calculated as |median_detected_ − median_non-detected_|/median_non-detected_ × 100%. Pathway names on the left are colored by KEGG Level 1 functional category, from top to bottom: dark blue for Metabolism, medium green for Organismal Systems, orange for Cellular Processes, purple for Genetic Information Processing, and dark red for Human Diseases.

Further stratified analyses revealed a clear gradient for *Limosilactobacillus*. Among *Limosilactobacillus*-positive samples, the number of pathways with decreased predicted abundance increased from none in T1 (0/27) to 15 in T2 (15/27) and 19 in T3 (19/27) (Table 2, Figure 2). Among these, 10 pathways were significant in both T2 and T3, including amino acid metabolism, cell growth and death, energy metabolism, metabolism of cofactors and vitamins, folding, sorting and degradation, and others (Table 2). In contrast, *Methylovirgula* showed no consistent pattern across tertiles, with no overlapping pathways (T1: 0/9, T2: 2/9, T3: 1/9), suggesting the associations may be weak or influenced by other factors. *Peptoclostridium* showed no significant associations in any group. No significant differences were observed in pathway abundances across the three groups (*p* > 0.05), indicating that genus–pathway covariations were confined to specific genera and pathways, while overall community function remained relatively stable.

### 3.5. Metal Index and Colorectal Polyp Progression

Ordinal logistic regression showed no significant association between the Metal Index and colorectal polyp progression (non-adenomatous benign lesions < non-advanced adenomas < advanced adenomas) (OR = 0.72, 95% CI: 0.484–1.072, *p* = 0.106). Combined with the microbial findings above, this result suggests that although the index was associated with specific genera and micro-level metabolic pathways, no corresponding differences were observed in colorectal polyp progression.

## 4. Discussion

In this study, we constructed a smoking-related multi-metal disruption index that captures the combined metal exposure effects, moving beyond the single-pollutant approach. We then examined its associations with gut microbial diversity, taxon colonization, metabolic pathways, and polyp progression in a population of colorectal polyp patients. The index showed dose-dependent increases in the detection rates of three genera, with two genera broadly associated with metabolic pathways but no significant associations with overall microbial diversity, global pathway abundance, or polyp progression. This hierarchical pattern suggests that smoking-related multi-metal exposure may induce early biological responses at the genus and pathway levels, even before clinical changes become detectable, highlighting the potential of gut microbiota as a sensitive indicator of early health risks associated with such exposure.

The normalized weights for As, Cd and Zn in the index were 0.496, 0.327, and 0.177, respectively. This distribution likely reflects differences in the sources and metabolic regulation of these three metals. As and Cd are non-essential heavy metals with no known biological function in humans and thus lack the homeostatic regulation mechanisms that exist for essential elements. They are major metal components of cigarette smoke, and smoking increases their inhalation, resulting in higher levels in smokers’ urine [9,10]. Smoking may also interfere with As methylation efficiency, altering its transformation and excretion patterns [13]. Zn, in contrast, is an essential trace element tightly regulated by metallothionein. Zn can be displaced by Cd, which has a much higher affinity for this protein [14,15], while the transfer rate of Zn from cigarette smoke (0.4–2.7%) is far lower than that of Cd (81–90%), and the amount contributed by smoke is negligible compared to daily dietary intake [16]. Thus, smoking affects Zn primarily through Cd-induced indirect disruption of Zn homeostasis rather than through direct increased intake. The concurrent elevation of these three metals reflects a systemic feature of smoking-related metal disruption: As through elevated exposure and altered metabolism, Cd through increased exogenous exposure, and Zn through homeostatic disruption.

Elevated urinary As and Cd reflect an increased circulatory burden. Once in the circulation, these metals can enter the intestinal lumen. Even at low concentrations, Cd can induce oxidative stress, inflammation, and intestinal bleeding; disrupt gut microbial homeostasis; and disturb energy, amino acid, lipid, nucleotide, and short-chain fatty acid metabolism [17]. The toxic mechanisms of As involve DNA damage and immunotoxicity [18], oxidative stress and intestinal barrier injury [19,20], epigenetic modifications [21,22], and disruption of bile acid metabolism [23]. In contrast, increased Zn excretion likely reflects Cd-induced disruption of Zn homeostasis and may place individuals at risk of a functional Zn deficiency. Zn deficiency, in turn, promotes oxidative stress and inflammation through Nrf2 inhibition and PI3K/Akt activation, thereby weakening antioxidant defenses [24]. These metal-related mechanisms may each contribute to disruption of the intestinal microenvironment. In our study, we observed pathways with decreased predicted abundance in T2 and T3, involving amino acid and energy metabolism, glycan biosynthesis and metabolism, cell growth and death, and protein folding, sorting and degradation. These changes are consistent with Cd and As toxicity and may also involve Zn disruption. Together, these findings suggest that the sustained decrease in the predicted abundance of these pathways may be associated with selective pressure from metal accumulation in the intestinal microenvironment.

Environmental pollution, including metal contamination, can reshape host-associated microbial communities and alter their functional profiles along contamination clines [25]. Microbial communities with sufficient resilience can resist such disturbances and initiate homeostatic responses to restore stability [26], and these responses may in turn create customized niches favoring the establishment of particular microbial populations [27]. One possible manifestation of these responses is the recruitment of taxa with complementary metabolic capacities. This colonization-based view may explain the higher relative abundance of *Limosilactobacillus* under metal exposure in our study. The functional attributes of *Limosilactobacillus* are also compatible with this view. Its genome encodes genes involved in amino acid, carbohydrate, cofactor, and vitamin metabolism [28], and these pathways overlap with those showing decreased predicted abundance under metal exposure. Experimental studies have further confirmed its ability to modulate the gut microbiota, exert antioxidant and anti-inflammatory effects, and enhance intestinal barrier integrity and immune regulation [29,30]. Animal studies have also shown that gut microbiota enriched in *Lactobacillus*, a genus within the same family as *Limosilactobacillus*, promoted adaptive stress-coping behaviors and preserved gut function under multifactorial stress conditions [31].

The stratified analysis further showed a pattern consistent with this colonization-based interpretation. In T1, no pathway differences were observed between detection-positive and detection-negative samples, suggesting that *Limosilactobacillus* colonization under low metal stress may represent a background level. At higher metal exposure levels, a graded increase in both detection rates and the number of pathways with decreased predicted abundance was observed, suggesting a dose-dependent pattern. This graded and persistent pattern is compatible with a compensatory process. The increase in *Limosilactobacillus* detection without corresponding changes in relative abundance suggests that its presence may be primarily colonization-based rather than proliferation-driven. *Limosilactobacillus* as a potential intervention target is supported by evidence that probiotics can mitigate heavy metal toxicity through biosorption, barrier enhancement, and immune modulation [32,33], although this possibility requires further experimental validation. In contrast to *Limosilactobacillus*, neither *Methylovirgula* nor *Peptoclostridium* exhibited a clear dose–response genus–pathway pattern. The absence of significant changes in overall community structure and clinical outcomes suggests that the current colonization shift may represent a compensatory state at the population level.

This study has several limitations. The cross-sectional design precludes causal inference, and the observed associations require further validation in longitudinal or experimental studies. The non-adenomatous benign lesions group had fewer subjects than the other polyp categories, which may limit the statistical power of analyses involving this group. Additionally, the absence of quantified smoking dose data (e.g., number of cigarettes per day or month) and detailed polyp characteristics (e.g., location, size, and number) limited our ability to assess smoking dose–response relationships and to fully characterize the association between metal exposure and polyp features. Despite these limitations, our findings offer a basis for identifying potential intervention targets, such as *Limosilactobacillus*, and generating testable hypotheses for future mechanistic investigations.

## 5. Conclusions

This study constructed a smoking-related multi-metal disruption index and examined its associations with the gut microbiota at multiple levels. The index showed significant dose-dependent increases in the detection rates of three genera, with *Limosilactobacillus* exhibiting the most robust pattern. Its detection rate was broadly negatively associated with the predicted abundance of pathways such as amino acid and energy metabolism. In stratified analyses, samples with *Limosilactobacillus* detection showed an increasing number of pathways with decreased predicted abundance as index levels rose. These findings suggest that smoking-related multi-metal disruption is associated with gut microbiota alterations in patients with CRA, a population with elevated cancer risk, involving reduced abundance of specific metabolic pathways and altered colonization of certain genera. This multi-Metal-Index approach provides a practical framework for assessing the health effects of complex environmental mixtures and highlights the potential of gut microbiota as a sensitive indicator of early exposure effects.

## Figures and Tables

**Table 1:** Detection rates and trends of gut genera across Metal Index tertiles.

Genera	Detection Rates, *n* (%)			Overall *p*	*p*_adj (T1 vs. T2)	*p*_adj (T1 vs. T3)	*p*_adj (T2 vs. T3)	*p* for Trend_adj
	T1 (*n* = 65)	T2 (*n* = 66)	T3 (*n* = 65)					
*Limosilactobacillus*	13 (19.7%)	23 (35.4%)	27 (41.5%)	0.022	0.084	0.016	0.574	0.027
*Methylovirgula*	9 (13.6%)	12 (18.5%)	23 (35.4%)	0.008	0.480	0.021	0.067	0.027
*Peptoclostridium*	4 (6.1%)	11 (16.9%)	15 (23.1%)	0.023	0.092	0.022	0.493	0.027
*Pygmaiobacter*	4 (6.1%)	0 (0.0%)	9 (13.8%)	0.006	0.163	0.163	0.009	0.138
[*Eubacterium*].brachy.group	27 (40.9%)	12 (18.5%)	24 (36.9%)	0.014	0.010	0.715	0.052	0.744
*Butyricimonas*	36 (54.5%)	20 (30.8%)	26 (40.0%)	0.021	0.021	0.157	0.390	0.141

**Table 2:** Stratified analysis of genus–pathway associations across Metal Index tertiles.

Genera	T1	T2	T3	Overlapping Pathways (T2 ∩ T3)
*Limosilactobacillus*	0/27	15/27	19/27	Amino acid metabolism; Biosynthesis of other secondary metabolites; Cell growth and death; Cellular community—prokaryotes; Endocrine and metabolic disease; Endocrine system; Energy metabolism; Folding, sorting and degradation; Glycan biosynthesis and metabolism; Metabolism of cofactors and vitamins
*Methylovirgula*	0/9	2/9	1/9	0
*Peptoclostridium*	0	0	0	0

Data are presented as the number of significant associations identified in each tertile/total number of KEGG Level 2 pathways significantly associated with each genus in the primary analysis. Significance was determined at *p*_adj < 0.05. Overlapping pathways (T2 ∩ T3) indicate pathways that were significant in both T2 and T3 groups. *Limosilactobacillus* had 10 overlapping pathways, while *Methylovirgula* and *Peptoclostridium* had none.

## Data Availability

The raw data supporting the conclusions of this article will be made available by the authors on request.

## Supplementary Materials

The following supporting information can be downloaded at https://www.mdpi.com/article/10.3390/toxics14100902/s1, Table S1: Baseline characteristics of participants by smoking status; Table S2: Multivariable logistic regression analysis of genus detection rates by Metal Index tertiles with progressive covariate adjustment.
