# Supplementary material for: Smoking-Related Multi-Metal Exposure Associated with Hierarchical Gut Microbiota Alterations in Colorectal Polyp Patients
Source: Toxics. 2026 Oct 12;14(10):902. doi: 10.3390/toxics14100902 (PMC13709704; doi:10.3390/toxics14100902)
Supplement: Supplementary file 1 [file toxics-14-00902-s001.zip › toxics-4537210-supplementary.pdf]

Supplementary Materials

Table S1. Baseline characteristics of participants by smoking status.

Variable	Never Smokers (n=138)	Current Smokers (n=34)	Former Smokers (n=24)	*P* Value
**1.Demographic characteristics**				
Age, years, median (IQR)	68 [61, 71]	67 [58, 68]	70 [65, 73.25]	**0.021**
Sex, male, n (%)	47 (34.1%)	31 (91.2%)	19 (79.2%)	**<0.001** [a]
BMI, kg/m^2, median (IQR)	23.55 [22.02, 25.48]	23.8 [21.15, 26.98]	24.2 [22.42, 26.08]	0.874
**2.Lifestyle factors, n (%)**				
Alcohol consumption, yes	10 (7.2%)	20 (58.8%)	6 (25.0%)	**0.001**
Dietary frequency				
Meat				
≥7 times/week	36 (26.1%)	10 (29.4%)	2 (8.3%)	
3–6 times/week	81 (58.7%)	20 (58.8%)	20 (83.3%)	0.215
≤2 times/week	21 (15.2%)	4 (11.8%)	2 (8.3%)	
Egg and dairy				
≥7 times/week	88 (63.8%)	14 (41.2%)	15 (62.5%)	
3–6 times/week	36 (26.1%)	14 (41.2%)	4 (16.7%)	0.066
≤2 times/week	14 (10.1%)	6 (17.6%)	5 (20.8%)	
Fruits and vegetables				
≥7 times/week	125 (90.6%)	30 (88.2%)	19 (79.2%)	
3–6 times/week	11 (8.0%)	4 (11.8%)	4 (16.7%)	0.301
≤2 times/week	2 (1.4%)	0 (0.0%)	1 (4.2%)	
Tea consumption, yes	73 (52.9%)	30 (88.2%)	15 (62.5%)	**0.001** [a]
**3.Urinary metals, median (IQR), μg/L**				
Sr	100.05 [58.90, 140.45]	144.30 [74.19, 223.35]	72.63 [60.96, 100.22]	**0.011**
As	22.94 [16.34, 39.71]	36.29 [20.52, 64.43]	19.56 [10.66, 31.83]	**0.016**
Zn	345.36 [211.75, 520.24]	525.93 [296.12, 758.96]	258.6 [148.03, 472.21]	**0.022**
Cd	0.96 [0.40, 1.97]	1.47 [0.78, 2.54]	0.65 [0.40, 1.48]	**0.031**
Fe	7.16 [0.55, 12.49]	11.96 [5.96, 19.34]	6.03 [0.55, 11.67]	0.051
Cu	10.19 [6.40, 15.99]	13.08 [8.93, 18.82]	9.00 [6.55, 12.45]	0.054
Se	27.89 [19.53, 43.29]	31.97 [23.78, 38.39]	18.67 [11.68, 37.32]	0.083
Rb	1607.04 [839.71, 2436.32]	1747.13 [945.56, 2939.47]	1048.42 [624.09, 1802.26]	0.122
Li	17.66 [13.09, 25.42]	21.65 [13.93, 30.98]	16.04 [9.62, 24.90]	0.130
Ba	1.35 [0.06, 2.47]	1.28 [1.05, 2.67]	1.08 [0.06, 1.55]	0.255
Cs	9.10 [5.77, 13.80]	10.52 [6.68, 17.62]	7.79 [5.21, 12.50]	0.340
Ni	1.62 [1.01, 2.43]	1.95 [1.19, 4.45]	1.42 [0.91, 2.84]	0.347
Sn	4.10 [2.51, 6.89]	4.65 [2.92, 6.03]	3.19 [1.57, 5.14]	0.358
Mn	0.05 [0.05, 0.05]	0.05 [0.05, 0.05]	0.05 [0.05, 0.05]	0.381
V	0.02 [0.02, 0.02]	0.02 [0.02, 0.02]	0.02 [0.02, 0.02]	0.437
U	0.00 [0.00, 0.00]	0.00 [0.00, 0.00]	0.00 [0.00, 0.00]	0.456
Pb	0.68 [0.30, 1.16]	0.76 [0.39, 1.53]	0.58 [0.27, 1.01]	0.578
Co	0.13 [0.00, 0.21]	0.11 [0.00, 0.25]	0.10 [0.00, 0.17]	0.643
Cr	0.05 [0.05, 0.05]	0.05 [0.05, 0.05]	0.05 [0.05, 0.05]	0.646
Tl	0.47 [0.22, 0.77]	0.55 [0.19, 0.77]	0.37 [0.10, 0.58]	0.675
Be	0.00 [0.00, 0.00]	0.00 [0.00, 0.00]	0.00 [0.00, 0.00]	0.750

Ga	0.02 [0.02, 0.02]	0.02 [0.02, 0.02]	0.02 [0.02, 0.02]	0.810
Al	0.69 [0.69, 13.18]	3.95 [0.69, 10.87]	0.69 [0.69, 20.44]	0.891
Mo	45.93 [26.51, 77.60]	44.33 [26.8, 71.78]	43.63 [25.49, 72.57]	0.931
Ag	0.00 [0.00, 0.00]	0.00 [0.00, 0.00]	0.00 [0.00, 0.00]	0.940
Sb	0.14 [0.01, 0.22]	0.14 [0.01, 0.22]	0.15 [0.01, 0.20]	0.995

[a] *P* value calculated using the chi-square test. All other categorical variables were analyzed using Fisher's exact test. Abbreviations: BMI, body mass index; IQR, interquartile range. Bold indicates statistical significance ($P < 0.05$). Data are presented as median (IQR) for continuous variables and n (%) for categorical variables.

Table S2. Multivariable logistic regression analysis of genus detection rates by metal index tertiles with progressive covariate adjustment.

Genus	Model	OR (95% CI), T2 vs T1	*P* Value	OR (95% CI), T3 vs T1	*P* Value
	Unadjusted	2.23 (1.01-4.93)	**0.047**	2.90 (1.33-6.33)	**0.008**
Limosilactobacillus	Smoking-adjusted	2.24 (1.01-4.94)	**0.046**	2.93 (1.31-6.54)	**0.009**
	Fully adjusted [a]	2.44 (1.09-5.46)	**0.030**	3.39 (1.49-7.76)	**0.004**
	Unadjusted	1.43 (0.56-3.68)	0.453	3.47 (1.46-8.26)	**0.005**
Methylovirgula	Smoking-adjusted	1.47 (0.57-3.80)	0.421	4.31 (1.76-10.60)	**0.001**
	Fully adjusted	1.50 (0.58-3.92)	0.406	4.67 (1.85-11.82)	**0.001**
	Unadjusted	3.16 (0.95-10.49)	0.061	4.65 (1.45-14.90)	**0.010**
Peptoclostridium	Smoking-adjusted	3.08 (0.92-10.33)	0.069	3.63 (1.10-11.98)	**0.035**
	Fully adjusted	3.11 (0.92-10.49)	0.068	3.70 (1.10-12.51)	**0.035**

[a] Fully adjusted for smoking, age, sex, BMI, alcohol consumption, and tea consumption. Abbreviations: OR: odds ratio; CI: confidence interval. Bold indicates statistical significance ($P < 0.05$).
